# COVID-Twitter-BERT: A natural language processing model to analyse COVID-19 content on Twitter

**DOI:** 10.3389/frai.2023.1023281

**Published:** 2023-03-14

**Authors:** Martin Müller, Marcel Salathé, Per E. Kummervold

**Affiliations:** ^1^Digital Epidemiology Lab, EPFL, Geneva, Switzerland; ^2^FISABIO-Public Health, Vaccine Research Department, Valencia, Spain

**Keywords:** natural language processing (NLP), COVID-19, language model (LM), BERT, text classification

## Abstract

**Introduction:**

This study presents COVID-Twitter-BERT (CT-BERT), a transformer-based model that is pre-trained on a large corpus of COVID-19 related Twitter messages. CT-BERT is specifically designed to be used on COVID-19 content, particularly from social media, and can be utilized for various natural language processing tasks such as classification, question-answering, and chatbots. This paper aims to evaluate the performance of CT-BERT on different classification datasets and compare it with BERT-LARGE, its base model.

**Methods:**

The study utilizes CT-BERT, which is pre-trained on a large corpus of COVID-19 related Twitter messages. The authors evaluated the performance of CT-BERT on five different classification datasets, including one in the target domain. The model's performance is compared to its base model, BERT-LARGE, to measure the marginal improvement. The authors also provide detailed information on the training process and the technical specifications of the model.

**Results:**

The results indicate that CT-BERT outperforms BERT-LARGE with a marginal improvement of 10-30% on all five classification datasets. The largest improvements are observed in the target domain. The authors provide detailed performance metrics and discuss the significance of these results.

**Discussion:**

The study demonstrates the potential of pre-trained transformer models, such as CT-BERT, for COVID-19 related natural language processing tasks. The results indicate that CT-BERT can improve the classification performance on COVID-19 related content, especially on social media. These findings have important implications for various applications, such as monitoring public sentiment and developing chatbots to provide COVID-19 related information. The study also highlights the importance of using domain-specific pre-trained models for specific natural language processing tasks. Overall, this work provides a valuable contribution to the development of COVID-19 related NLP models.

## 1. Introduction

Twitter has been a valuable source of news and a public medium for expression during the COVID-19 pandemic. However, manually classifying, filtering, and summarizing the large amount of information available on COVID-19 on Twitter is impossible and has also been a challenging task to solve with tools from the field of machine learning and natural language processing (NLP). To improve our understanding of Twitter messages related to COVID-19 content as well as the analysis of this content, we have therefore developed a model called COVID-Twitter-BERT (CT-BERT).^1^

Transformer-based models have changed the landscape of NLP. Models such as BERT, RoBERTa, and ALBERT are all based on the same principle – training bi-directional transformer models on huge unlabeled text corpuses (Vaswani et al., 2017; Devlin et al., 2018; Lan et al., 2019; Liu et al., 2019). This process is done using methods such as mask language modeling (MLM), next sentence prediction (NSP), and sentence order prediction (SOP). Different models vary slightly in how these methods are applied, but in general, all training is done in a fully unsupervised manner. This process generates a general language model that is then used as input for a supervised finetuning for specific language processing tasks, such as classification, question-answering models, and chatbots.

Our model is based on the BERT-LARGE (English, uncased, whole word masking) model. BERT-LARGE is trained mainly on raw text data from Wikipedia (3.5B words) and a free book corpus (0.8B words) (Devlin et al., 2018). Whilst this is an impressive amount of text, it still contains little information about any specific subdomain. To improve performance in subdomains, we have seen numerous transformer-based models trained on specialized corpuses. Some of the most popular ones are BIOBERT (Lee et al., 2020) and SCIBERT (Beltagy et al., 2019). These models are trained using the exact same unsupervised training techniques as the main models (MLM/NSP/SOP). They can be trained from scratch, but this requires a very large corpus, so a more common approach is to start with the trained weights from a general model. In this study, this process is called domain-specific pretraining. When trained, such models can be used as replacements for general language models and be trained for downstream tasks.

## 2. Method

The CT-BERT model is trained on a corpus of 160M tweets about the coronavirus collected through the Crowdbreaks platform (Müller and Salathé, 2019) during the period from January 12 to April 16, 2020. Crowdbreaks uses the Twitter filter stream API to listen to a set of COVID-19-related keywords^2^ in the English language. These downloaded tweets are the basis for creating the training corpus. Prior to training, the original corpus was cleaned for retweet tags. Each tweet was pseudonymised by replacing all Twitter usernames with a common text token. A similar procedure was performed on all URLs to web pages. We also replaced all unicode emoticons with textual ASCII representations (e.g., :smile: for ☺) using the Python emoji library.^3^ Starting from the 160M tweets, all retweets, duplicates and close duplicates were removed. This resulted in a final corpus of 22.5M tweets that comprise a total of 0.6B words. The domain-specific pretraining dataset therefore consists of 1/7th the size of what is used for training the main base model. Tweets were treated as individual documents and segmented into sentences using the spaCy library (Honnibal and Montani, 2017).

All input sequences to the BERT models are converted to a set of tokens from a 30.000-word vocabulary. As all Twitter messages are limited to 280 characters, this allows us to reduce the sequence length to 96 tokens, thereby increasing the training batch sizes to 1.024 examples. We use a dupe factor of 10 on the dataset, resulting in 285M training examples and 2.5M validation examples. A constant learning rate of 2e-5, as recommended on the official BERT GitHub^4^ when doing domain-specific pretraining.

Loss and accuracy was calculated through the pretraining procedure. For every 100.000 training steps, we therefore save a checkpoint and finetune this toward a variety of downstream classification tasks. Distributed training was performed using Tensorflow 2.2 on a TPU v3-8 (128GB of RAM) for 120 h.

All scores in this work have been computed using the scikit-learn Python package. Throughout this work, F1 scores are using macro averaging.

### 2.1. Evaluation

To assess the performance of our model on downstream classification tasks, we selected five independent training sets (see Table 1). Three of them are publicly available datasets, and two are from internal projects not yet published. All datasets consist of Twitter-related data.

**Table 1:** Overview of the evaluation datasets.

**Dataset**	**Classes**	**Train**	**Dev**	**Labels**
COVID-19 Category (CC)	2	3,094	1.031	Personal		News	
Vaccine Sentiment (VC)	3	5.000	3.000	N	Neutral		Positive	
Maternal Vaccine Stance (MVS)	4	1.361	817	Disc		A		N		Promotional	
Stanford Sentiment Treebank 2 (SST-2)	2	67.349	872	Negative		Positive	
Twitter Sentiment SemEval (SE)	3	6.000	817	Neg		Neutral		Positive	

All five evaluation datasets are multi-class datasets with sometimes strong label imbalance, visualized by the proportional bar width in the label column. N and Neg stand for negative; Disc and A stand for discouraging and ambiguous, respectively.

#### 2.1.1. COVID-19 category (CC)

This is an internal dataset of tweets that are sampled from the training dataset for CT-BERT, specifically for the period between January 12 and February 24, 2020. Annotators on Amazon Turk (MTurk) were asked to categorize a given tweet text into either being a personal narrative (33.3%) or news (66.7%). The annotation was performed using the Crowdbreaks platform (Müller and Salathé, 2019).

#### 2.1.2. Vaccine sentiment (VS)

This dataset contains a collection of measles- and vaccination-related US-geolocated tweets collected between March 2, 2011 and October 9, 2016. The dataset was first used by Pananos et al. (2017), but a modified version from Müller and Salathé (2019) was used here. The dataset contains three classes: positive (toward vaccinations) (51.9%), negative (7.1%), and neutral/others (41.0%). The neutral category was used for tweets which are either irrelevant or ambiguous. Annotation was performed on MTurk.

#### 2.1.3. Maternal vaccine stance (MVS)

The dataset is from a project related to the stance toward the use of maternal vaccines (Kummervold et al., 2021). The methodology for creating the dataset is also explained in Martin et al. (2020). Experts in the field annotated the data into four categories: neutral (41.0%), discouraging (25.3%), promotional (43.9%), and ambiguous (14.3%). Each tweet was annotated 3-fold, and disagreement amongst the experts was resolved in each case by using a common scoring criterion.

#### 2.1.4. Twitter sentiment SemEval (SE)

This is an open dataset from SemEval-2016 Task 4: Sentiment Analysis in Twitter (Nakov et al., 2019). In particular, we used the dataset for subtask A, a dataset annotated 5-fold into three categories: negative (15.7%), neutral (45.9%), and positive (38.4%). We make a small adjustment to this dataset by fully anonymizing links and usernames.

#### 2.1.5. Stanford sentiment treebank 2 (SST-2)

SST-2 is a public dataset consisting of binary sentiment labels, negative (44.3%) and positive (55.7%), within sentences (Socher et al., 2013). Sentences were extracted from a dataset of movie reviews (Pang and Lee, 2005) and did not originate from Twitter, making SST-2 our only non-Twitter dataset.

The division of the dataset into subsets for the SST-2 and SE datasets was pre-determined. In the case of the other datasets, our aim was to divide the data roughly 30–50% for the training and validation sets, respectively, with the remaining 20% designated as the test set, which was not used in this study. The validation set was the only subset used in this research.

Our intention was not to optimize the finetuned models but to thoroughly evaluate the performance of the domain-specific CT-BERT-model. We experimented with different numbers of epochs for each training dataset for BERT-LARGE (i.e., checkpoint 0 of CT-BERT) and selected the optimal one. We then used this number in subsequent experiments on the respective dataset. We ended with three epochs for SST-2 (291 steps), CC (291 steps) and SE (564 steps), five epochs for VC (780 steps), and 10 epochs for MVS (430 steps), all with a learning rate of 2e-05 and a batch size of 32. The number of epochs was dependent on both the size and balance of the categories. Larger and unbalanced sets require more epochs.

## 3. Results

### 3.1. Domain-sepcific pretraining

**Figure 1:**
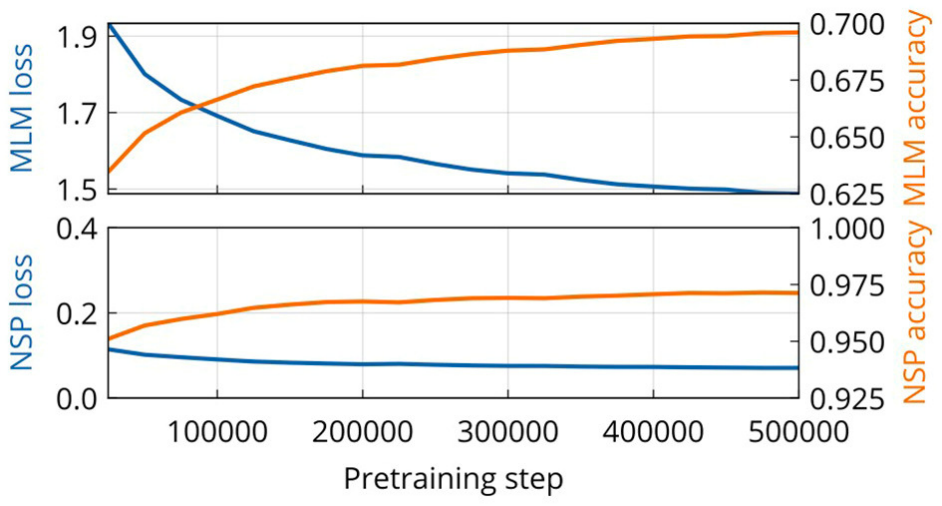
Evaluation metrics for the domain-specific pretraining of CT-BERT. Shown are the loss and accuracy of masked language modeling (MLM) and next sentence prediction (NSP) tasks.

Figure 1 shows the progress of pretraining CT-BERT at intervals of 25k training steps and the evaluation of 1k steps on a held-out validation dataset. All metrics considered improve throughout the training process. The improvement on the MLM loss task is most notable and yields a final value of 1.48. The NSP task improves only marginally, as it already performs very well initially. Training was stopped at 500.000, an equivalent of 512M training examples, which we consider as our final model. This corresponds to roughly 1.8 training epochs. All metrics for the MLM and NLM tasks improve steadily throughout training. However, using loss/metrics for these tasks to evaluate the correct time to stop training is difficult.

### 3.2. Evaluation on classification datasets

To assess the performance of our model properly, we compared the mean F1 score of CT-BERT with that of BERT-LARGE on five different classification datasets. We adapted the number of training epochs for each dataset according to its size in order to have a similar number of training steps for each dataset. Our final model shows higher performance on all datasets (a mean F1 score of 0.833) compared with BERT-LARGE (a mean F1 score of 0.802). As the initial performance varies widely across datasets, we compute the relative improvement in marginal performance (ΔMP) for each dataset. ΔMP is calculated as follows:


$$\begin{array}{l} \Delta \text{MP}=\frac{F_{1,\text{CT-BERT}}-F_{1,\text{BERT-LARGE}}}{1-F_{1,\text{BERT-LARGE}}} \end{array}$$

From Table 2 we observe that the largest improvement of our model on the COVID-19-specific dataset (CC), with a ΔMP value of 25.88%. The marginal improvement is also high on the Twitter datasets related to vaccine sentiment (MVS). Our model likewise shows some improvements on the SST-2 and SemEval datasets, but to a smaller extent.

**Table 2:** Comparison of the final model performance with BERT-LARGE.

**Dataset**	**BERT-LARGE**	**CT-BERT**	**ΔMP**
COVID-19 Category (CC)	0.931	0.949	25.88%
Vaccine Sentiment (VC)	0.824	0.869	25.27%
Maternal Vaccine Stance (MVS)	0.696	0.748	17.07%
Stanford Sentiment Treebank 2 (SST-2)	0.937	0.944	10.67%
Twitter Sentiment SemEval (SE)	0.620	0.654	8.97%
Average	0.802	0.833	17.57%

CT-BERT shows improvements on all datasets. The marginal improvement is the highest on the COVID-19-related dataset (CC) and lowest on the SST-2 and SemEval datasets.

### 3.3. Evaluation on intermediary pretraining checkpoints

**Figure 2:**
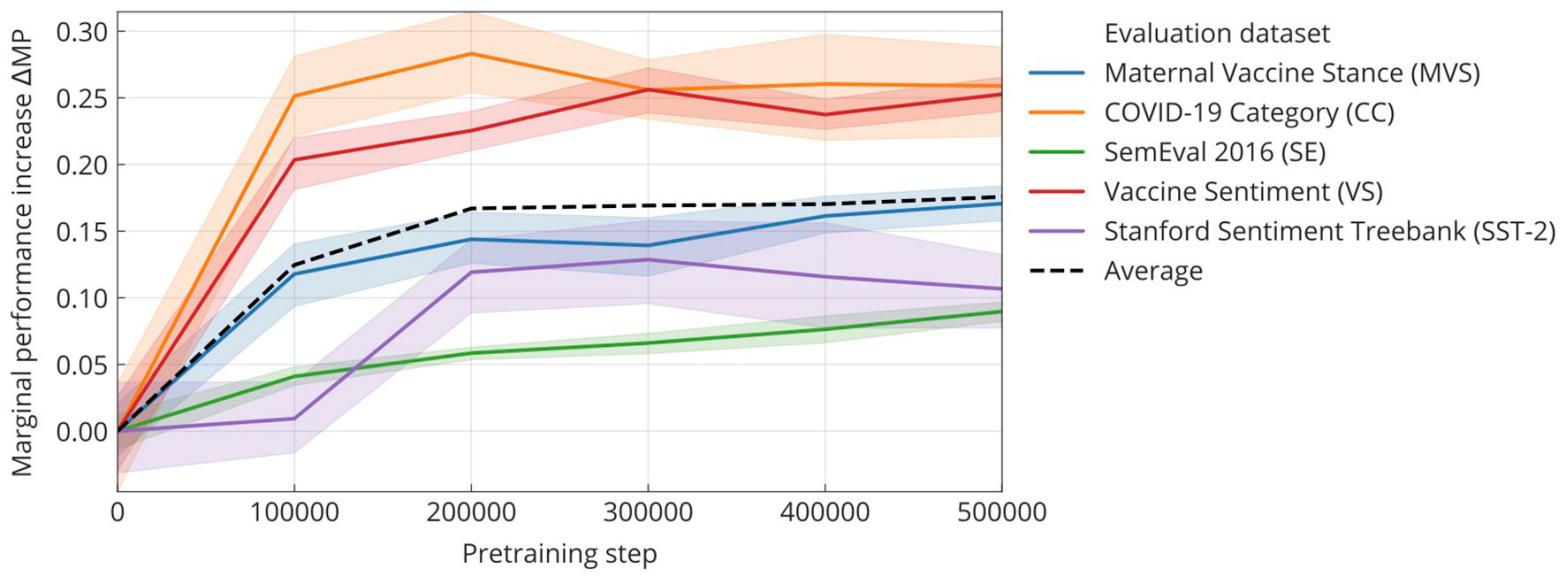
Marginal performance increase in the F1 score (ΔMP) on finetuning on various classification tasks at increasing steps of pretraining. Zero on the x-axis corresponds to the base model, which is BERT-LARGE in this case. Our model improves on all evaluated datasets, with the biggest relative improvement being in the COVID-19 category dataset. The bands show the standard error of the mean (SEM) out of 10 repeats. From this we can also estimate the standard deviation $\text{SD}=\text{SEM}*\sqrt{10}$.

So far, we have seen improvements in the final CT-BERT model on all evaluated datasets. To understand whether the observed decrease in loss during pretraining linearly translates into performance on downstream classification tasks, we evaluated CT-BERT on five intermediary versions (checkpoints) of the model and on the zero checkpoint, which corresponds to the original BERT-LARGE model. At each intermediary checkpoint, 10 repeated training runs (finetunings) for each of the five datasets were performed, and the mean F1 score was recorded. Figure 2 shows the marginal performance increase (ΔMP) at specific pretraining steps. Our experiments show that downstream performance increases fast up to step 200k in the pretraining and only demonstrates marginal improvement afterwards. The loss curve, on the other hand, shows a gradual increase even after step 200k. We also note that for the COVID-19-related dataset, most of the marginal improvement occurred after 100k pretraining steps. SST-2, the only non-Twitter dataset, improves much more slowly and reaches its final performance only after 200k pretraining steps.

Amongst runs on the same model and dataset, some degree of variance in performance was observed. This variance is mostly driven by runs with a particularly low performance. We observe that the variance is dataset dependent, but it does not increase throughout different pretraining checkpoints and is comparable to the variance observed on BERT-LARGE (pretraining step zero). The most stable training seems to be on the SemEval training set, and the least stable one is on SST-2, but most of this difference is within the error margins.

## 4. Discussion

The most accurate way to evaluate the performance of a domain-specific model is to apply it on specific downstream tasks. CT-BERT is evaluated on five different Twitter-based datasets. Compared to BERT-LARGE, it improves significantly on all datasets. However, the improvement is largest in datasets related to health, particularly in datasets related to COVID-19. We therefore expect CT-BERT to perform similarly well on other classification problems on COVID-19-related data sources, but particularly on text derived from social media platforms.

Whilst it is expected that the benefit of using CT-BERT instead of BERT-LARGE is greatest when working with Twitter COVID-19 text, it is reasonable to expect some performance gains even when working with general Twitter messages (SemEval dataset) or with a non-Twitter dataset (SST-2).

Our results show that the MLM and NSP metrics during the pretraining align to some degree with downstream performance on classification tasks. However, compared with COVID-19 or health-related content, out-of-domain text might require longer pretraining to achieve a similar performance boost.

Whilst we have observed an improvement in performance on classification tasks, we did not test our model on other natural language understanding tasks. Furthermore, at the time of this paper's writing, we only had access to one COVID-19-related dataset. The general performance of our model might be improved further by considering pretraining under different hyperparameters, particularly modifications to the learning rate schedules, training batch sizes and optimizers. Future work might include evaluation on other datasets and the inclusion of more recent training data.

The best way to evaluate pretrained transformer models is to finetune them on downstream tasks. Finetuning a classifier on a pre-trained model is considered computationally cheap. The training time is usually done in an hour or two on a GPU. Using this method for evaluation is more expensive, as it requires evaluating multiple checkpoints to monitor improvement and on several varied datasets to show robustness. As finetuning results vary between each run, each experiment must be performed multiple times when the goal is to study the pretrained model. In this case, we repeated the training for six checkpoints, 10 runs for each checkpoint on all the five datasets. A total of 300 evaluation runs were performed. The computational cost for evaluation is therefore on par with the pretraining. Large and reliable validation sets make this task easier, as the number of repetitions can be reduced.

All the tests are done on categorization tasks, as this task is easier in terms of both data access and evaluation. However, transformer-based models can be used for a wide range of tasks, such as named entity recognition and question answering. It is expected that CT-BERT can also be used for these kinds of tasks within our target domain.

Our primary goal in this work was to obtain stable results on the finetuning in order to evaluate the pre-trained model, not to necessarily optimize the finetuning. The number of finetuning epochs and the learning rate are, for instance, have been optimized for BERT-LARGE, not for CT-BERT. This means that there is still great room for optimization on the downstream task.

## 5. Limitations

In this study, we demonstrate that our model outperforms BERT Large, which at the time of the study held the state-of-the-art performance for many NLP tasks. However, it should be noted that since the completion of our study, several other language models have been introduced that may surpass the performance of BERT Large and potentially also CT-BERT. Nevertheless, the methodology used to improve BERT Large in the development of CT-BERT could potentially be applied to other models in order to achieve similar performance improvements.

## Data availability statement

The model, code, and public datasets are available in our GitHub repository: https://github.com/digitalepidemiologylab/covid-twitter-bert.

## Author contributions

All authors listed have made a substantial, direct, and intellectual contribution to the work and approved it for publication.

## Footnotes

^1^ https://github.com/digitalepidemiologylab/covid-twitter-bert

^2^wuhan, ncov, coronavirus, covid, SARS-CoV-2.

^3^ https://pypi.org/project/emoji/

^4^ https://github.com/google-research/bert
